# Mutations in virus-derived small RNAs

**DOI:** 10.1038/s41598-020-66374-2

**Published:** 2020-06-12

**Authors:** Deepti Nigam, Katherine LaTourrette, Hernan Garcia-Ruiz

**Affiliations:** Department of Plant Pathology and Nebraska Center for Virology, University of Nebraska-Lincoln, Nebraska, United States of America

**Keywords:** Non-coding RNAs, RNAi

## Abstract

RNA viruses exist as populations of genome variants. Virus-infected plants accumulate 21–24 nucleotide small interfering RNAs (siRNAs) derived from viral RNA (virus-derived siRNAs) through gene silencing. This paper describes the profile of mutations in virus-derived siRNAs for three members of the family *Potyviridae*: *Turnip mosaic virus* (TuMV), *Papaya ringspot virus* (PRSV) and *Wheat streak mosaic virus* (WSMV). For TuMV in *Arabidopsis thaliana*, profiles were obtained for mechanically inoculated rosette leaves and systemically infected cauline leaves and inflorescence. Results are consistent with selection pressure on the viral genome imposed by local and systemic movement. By genetically removing gene silencing in the plant and silencing suppression in the virus, our results showed that antiviral gene silencing imposes selection in viral populations. Mutations in siRNAs derived from a PRSV coat protein transgene in the absence of virus replication showed the contribution of cellular RNA-dependent RNA polymerases to the generation of mutations in virus-derived siRNAs. Collectively, results are consistent with two sources of mutations in virus-derived siRNAs: viral RNA-dependent RNA polymerases responsible for virus replication and cellular RNA-dependent RNA polymerases responsible for gene silencing amplification.

## Introduction

In plants virus infection triggers antiviral gene silencing that results in the accumulation of virus-derived small interfering RNAs (siRNAs) that are normally 21–24 nucleotides long and associate with argonaute proteins to form RNA-induced silencing complexes and guide translational repression or cleave of viral RNA targets based on sequence complementarity^1–3^. Virus-derived siRNAs are generated by dicer-like proteins (DCL), mainly DCL4 (21-nt) and DCL2 (22-nt), using dsRNA substrates that include virus replication intermediates and fold-back structures in single stranded RNA^4–7^. Additionally, siRNAs are silencing signals and move cell-to-cell, root to shoot, and in some cases between plants and fungi^8^.

Plant virus infection depends on the ability of the virus to replicate in infected cells, move cell-to-cell via plasmodesmata and systemically^9^. Genetic heterogeneity in combination with genetic robustness allows viruses to adapt to diverse hosts, vectors, and environments^10–12^. In infected plants, viruses exists as diverse populations of genomes forming a quasispecies^12^. A critical source of variation that drives virus evolution is the viral RNA-dependent RNA polymerases which lack proof-reading activity and introduce mutations (nucleotide substitutions, insertions, or deletions) during virus replication. As a result, each replication cycle might produce new variants containing nucleotide substitutions, insertions, or deletions^11–15^. Selection eliminates unfit variants and favors variants with a competitive advantage, resulting in one or few dominant genetic variants surrounded by a large number of low-frequency, less-fit, or emerging variants^12,15,16^.

High-throughput sequencing has expanded our knowledge of virus-derived siRNA biogenesis, diversity, virus-host interactions, their effect on host gene expression, virus discovery, and diagnostics^17–24^. Using computational approaches, complete genomes of DNA or RNA viruses have been assembled *de novo* using virus-derived siRNAs^21,25,26^, and viral variants were detected^25^, suggesting that variation in virus-derived siRNAs reflect variation in genomic RNA. However, nucleotide variation in virus-derived siRNA has not been characterized. Here, we profiled virus-derived siRNA variation in three members of the *Potyviridae*: *Turnip mosaic virus* (TuMV), *Papaya ringspot virus* (PRSV) and *Wheat streak mosaic virus* (WSMV).

Virus-derived siRNAs measuring 21-to 24-nt were used to profile mutations in several tissues or in cultivars with contrasting tolerance to virus infection. Small RNA profiles were generated for TuMV-infected *Arabidopsis thaliana* (Arabidopsis) ecotype Colombia, and comparisons were made between the mechanically inoculated rosette leaves, and systemically infected cauline leaves, or inflorescence. As they move across tissues, viruses face several selection pressures. In the collection of Arabidopsis single mutants available, only *ago2-1* meets the requirements of being systemically infected by suppressor-deficient TuMV-AS9 and not preventing the biogenesis of virus-derived siRNAs^7,27^. Thus, to determine the effect of antiviral gene silencing on the accumulation of mutations, we partially removed gene silencing in the host by using single *ago2–1* Arabidopsis mutant plants. In the virus, we genetically removed silencing suppression by using suppressor deficient TuMV-AS9^27^. Accumulation of mutations in virus-derived siRNAs is in agreement with antiviral gene silencing imposing selection pressure in viral populations.

In plants, amplification of gene silencing is necessary to establish an antiviral state and a significant proportion of virus-derived siRNA is amplification dependent^7,28,29^. A subset of the viral RNA population targeted by siRNAs is used as a template by cellular by RNA-dependent RNA polymerases to synthesize dsRNA that is processed into secondary virus-derived siRNAs by DCL proteins^3,7,29^. This biogenesis pathway predicts that cellular RNA-dependent RNA polymerases contribute to the generation of mutations in virus-derived siRNAs. To test this hypothesis, we profiled siRNAs derived from a PRSV coat protein transgene, in the absence of PRSV replication. Results support the contribution of cellular RNA-dependent RNA polymerases to the generation of mutations in virus-derived siRNAs.

## Results

### Single nucleotide polymorphisms in virus-derived siRNAs

**Figure 1:**
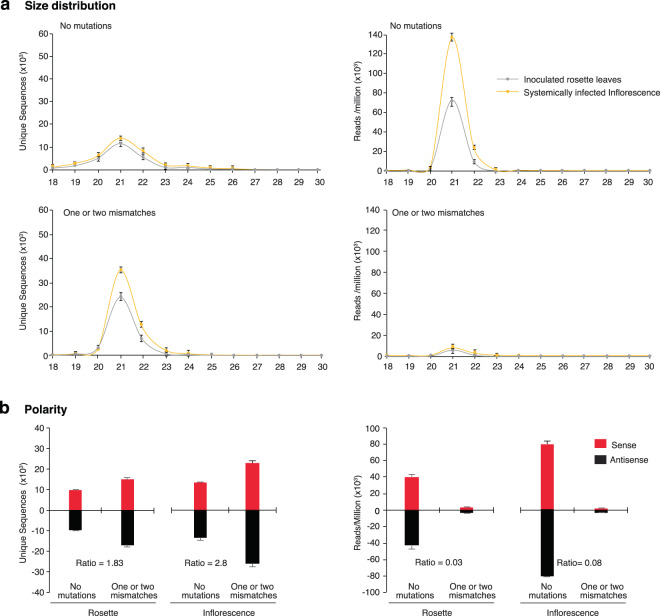
Profile of TuMV-derived siRNAs in wild type *Arabidopsis thaliana*. Values are average and standard error from two biological replicates. Abundance was normalized to reads per million. Mechanically inoculated rosette leaves were sampled at 7 days post inoculation (dpi). Samples from systemically infected inflorescence were collected at 10 dpi. (**a**) Size distribution and abundance of 18–30 nt unique sequences with no mutations, and sequences with one or two mismatches. (**b**) Polarity and abundance by tissue and number of mismatches. Only 21- to 24-nt siRNAs were included. Te ratio of siRNAs with mismatchest to siRNAs with no mutations is indicated for each tissue.

We used TuMV-derived siRNAs to identify and profile mutations in virus-derived siRNA. As described for multiple virus-host combinations^17,30,31^, TuMV-derived siRNA are predominantly 21- to 24-nt long. With respect to the TuMV reference genome, virus-derived siRNAs were classified into two groups: perfect match (zero mutations), and one or two mismatches (nucleotide insertion, deletions or substitutions). Sequences with more than two mismatches represented less than 1% and were not included (Fig. 1).

To characterize populations of virus-derived siRNAs we used two parameters: number of unique sequences, and the number of times a unique sequence (abundance) was detected. Abundance was normalized to reads per million^7,32^. In virus-derived siRNAs, mismatches to the reference genome might represent bona fide mutations or sequencing errors. High-throughput sequencing of virus-derived siRNAs has an error rate of 0.0079 per nucleotide and 0.000053 per read^7^. Based on this frequency, in a population of 52,000 unique 21-nt long sequences with one mismatch, 2.7 unique sequences might result from sequencing error (Fig. 1a). Accordingly, mismatches to the reference genome represent bona fide mutations in virus-derived siRNAs.

Unique virus-derived siRNAs with no mutations were less frequent (2 to 4-fold) than unique siRNAs with one or two mutations. In contrast, reads of siRNAs with no mutations were more abundant (14 to 16-fold) than siRNAs with mutations (Fig. 1a). This pattern suggests that mutations are introduced at a high frequency in a small fraction of viral RNA and are not fixed in the viral genome. Instead, mutations might be introduced during RNA synthesis of a template that is not replicating, such as dsRNA generated by cellular RNA-dependent RNA polymerases.

### There is no polarity bias in TuMV-derived siRNAs

For positive-strand RNA viruses, in infected cells, the positive strand RNA is 10 to 100 times more abundant than negative-strand RNA. Such discrepancy is mainly due to positive-strand RNA in virions. In the absence of virion formation, positive-strand RNA accumulates two to four times more than the negative-strand RNA^33–37^.

TuMV is a positive strand RNA virus that replicates through dsRNA intermediates^38^. Abundance of TuMV genomic RNA predicts that virus-derived siRNAs of positive polarity are more abundant than virus-derived siRNAs of negative polarity. However, consistent with results previously described for TuMV^7,27^ and other positive-strand RNA viruses^17,30,31,39^, in the analyses describe here, TuMV-derived siRNAs of positive and negative polarity were equally abundant (Fig. 1b). The lack of strand bias in the accumulation of virus-derived siRNAs suggests that cellular RNA-dependent RNA polymerases introduce mutations during the synthesis of viral dsRNA necessary for gene silencing amplification.

### Systemic movement imposes selection pressure in TuMV

Viral RNA-dependent RNA polymerases constantly create new variants during replication, because they have no proofreading activity^13,14^. Mutations introduced in the genome early in infection might be eliminated by selection or fixed and propagated through replication and movement. These scenarios predict that early in infection, mutations are more abundant in locally infected leaves than in systemically infected tissues. To test this hypothesis, we compared the profile of TuMV-derived siRNAs in mechanically inoculated Arabidopsis rosette leaves to those in systemically infected inflorescence. In mechanically inoculated rosette leaves, the number of unique virus-derived siRNAs with one or two mutations was 1.83 times higher than the number of unique siRNAs without mutations. In systemically infected inflorescence of the same plants, the number of unique virus-derived siRNA with one or two mutations was 1.6 times higher than the number of unique virus-derived siRNAs without mutations (Fig. 1b). In both tissues, virus-derived siRNAs with no mutations were more abundant than virus-derived siRNAs with mutations. The ratios were 0.03 and 0.08 in mechanically inoculated rosette leaves and in systemically infected inflorescence, respectively (Fig. 1b). Accordingly, early in infection mutations are more abundant and the viral population is more diverse than in systemically infected tissue, consistent with cell-to-cell and systemic movement imposing selection pressure on virus populations. These differences are likely an underestimation because, as indicated below, cellular RNA-dependent RNA polymerases contribute to the generation of mutations detected in virus-derived siRNAs.

### Asymmetry in the genomic distribution of mutations

**Figure 2:**
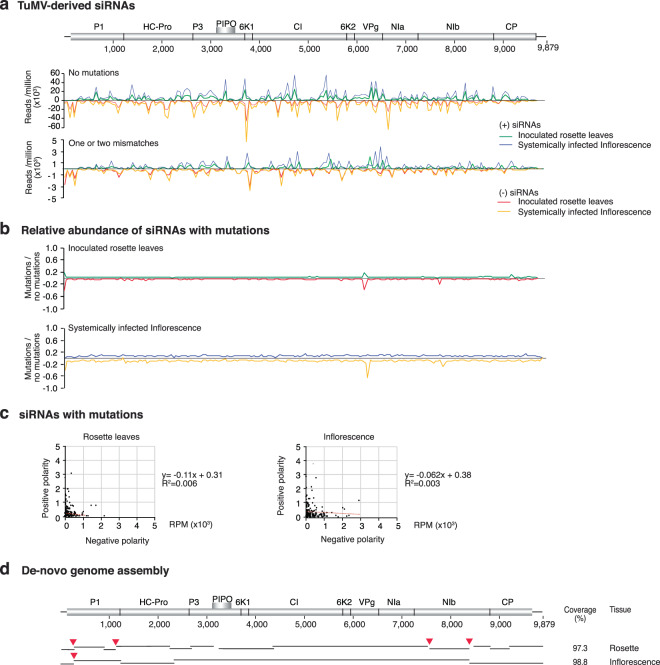
Genome-wide distribution of TuMV-derived siRNAs in wild type *Arabidopsis thaliana* and *de-novo* assembly of the TuMV genome. 21- to 24-nt virus-derived siRNAs were pooled and correspond to samples in Fig. 1. Abundance was normalized to reads per million. (**a**) Genome-wide distribution of TuMV-derived siRNAs by match class in inoculated rosette leaves and in systemically infected inflorescence. (**b**) Abundance of siRNAs with one or two mismatches relative to siRNAs with no mutations. (**c**) Relationship between siRNAs of positive and negative polarity and containing mutations. (**d**) *De-novo* assembly of the TuMV genome using virus-derived siRNAs without mutations. Lines represent contigs and red arrowheads point to gaps in the assembly. In this TuMV clone, HC-Pro is marked with 6xHIS at the N terminus^27^.

Viral replication and gene silencing amplification are mediated by the viral replicase and by cellular RNA-dependent RNA polymerases, respectively. Based on this framework, in substrates produced by the viral replicase, mutations in virus-derived siRNAs map to the same coordinates in the positive and negative strand. In contrast, in substrates produced by cellular RNA-dependent RNA polymerases, mutations in virus-derived siRNAs from the positive strand map to different areas of the genome respect to those derived from the negative strand. To test this hypothesis, we mapped the distribution of virus-derived siRNAs with and without mutations (Fig. 2a). Genome-wide and correlation plots were generated between siRNAs that mapped to positive and negative strand in inoculated rosette leaves and in systemically infected inflorescence (Fig. 2b,c). In mechanically inoculated rosette leaves and systemically infected inflorescence, the distribution of mutations along the viral genome is different for the positive and the negative strand (Fig. 2b,c). The asymmetry in the genomic distribution of mutations supports the hypothesis that part of the mutations in virus-derived siRNAs is introduced by cellular RNA-dependent RNA polymerases.

In an independent test of the same hypothesis, we assembled the TuMV genome from virus-derived siRNAs without mutations. Contigs covered 97.3% and 98.8% of the genome in samples from locally infected rosette leaves and systemically infected inflorescence, respectively (Fig. 2d). In samples from mechanically inoculated rosette leaves, four gaps were detected, two in the P1 cistron and two in the NIb cistron. In contrast, in systemically infected inflorescence, only one gap was detected along P1 and colocalized with one of the gaps observed in rosette leaves (Fig. 2d). Gaps in the assembled genome might be due to a lack of virus-derived siRNAs or to a high abundance of mutations. Genome-wide distribution of the ratio of siRNAs with mutations to siRNAs without mutations showed that gaps in the assembly were not due to lack of virus-derived siRNAs (Fig. 2b). These results are consistent with the viral population being more diverse early in infection in inoculated leaves than in systemically infected tissue.

### Tissue-specific mutations

**Figure 3:**
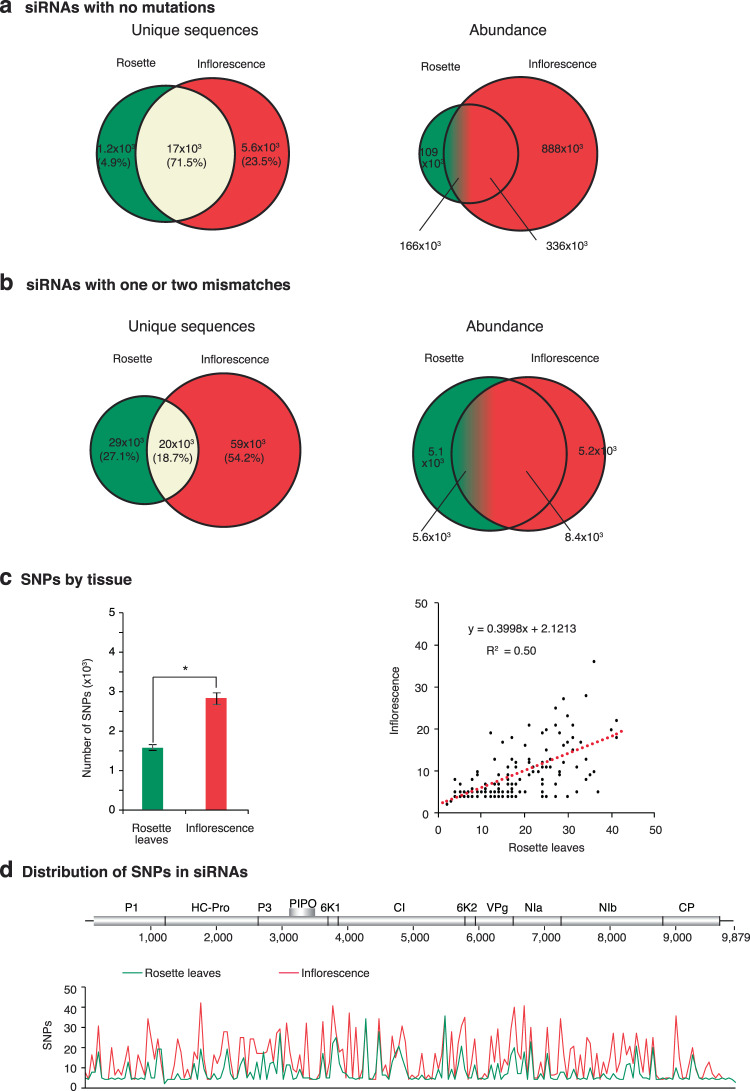
Comparison of TuMV-derived siRNAs in inoculated rosette leaves and systemically infected inflorescence. 21- to 24-nt siRNAs were pooled and correspond to samples in Fig. 1. Unique sequences were counted per tissue and their abundance normalized to reads per million. Values are the average of two biological replicates. Virus-derived siRNAs with (**a**) no mutations and (**b**) with one or two mismatches. (**c**) Accumulation of single nucleotide polymorphisms (SNPs) in virus-derived siRNAs in inoculated rosette leaves, in systemically infected inflorescence, and their correlation. The asterisk (*) indicates significant difference (p-value < 0.001). (**d**) Genome-wide distribution of single nucleotide polymorphisms in virus-derived siRNAs estimated in a 50-nt window.

Mutations introduced early in infection may be removed by purifying selection, or fixed and propagated in systemically infected tissue. Additionally, in systemically infected tissue new mutations might originate. To test this hypothesis, we separated mutations by tissue (Fig. 3). Unique sequences with no mutations were more diverse and more abundant in systemically infected inflorescence than in mechanically inoculated rosette leaves (Fig. 3a). This is related to the higher accumulation of virus in systemically infected inflorescence than in inoculated rosette leaves^27^. Interestingly, while 71.5% of the unique sequences were common between leaves and inflorescences, their abundance was 2-fold higher in the systemically infected inflorescence. The proportion of unique sequences with mutations (54.2%) in systematically infected inflorescence tissue was 2.03 times higher than in inoculated rosette leaves (27.1%) while the common mutations were only 18.7% and their abundance represented 73% of the observe in inflorescences (Fig. 3b).

To characterize tissue-specific mutations in virus-derived siRNAs, we quantified and mapped single nucleotide polymorphisms (SNP) in TuMV-derived siRNAs from mechanically inoculated rosette leaves and systemically infected inflorescence (Fig. 3c,d). The total number of single nucleotide polymorphisms was higher in systemically infected inflorescence than in locally inoculated rosette leaves (Fig. 3c), which is consistent with the higher accumulation of virus in systemically infected inflorescence than in inoculated rosette leaves^27^. In TuMV-derived siRNAs, single nucleotide polymorphisms accumulated in all parts of the genome both in mechanically inoculated rosette leaves and in systemically infected inflorescence, and there was no correlation between the genome-wide distribution of mutations across tissues (Fig. 3c,d).

Together, these results support the hypothesis that a high proportion of mutations that originate early in infection are lost in the population that establishes systemic infection, only a small proportion of mutations might be fixed in the virus genome, and that new mutations arise in systemically infected tissue.

### Virus-derived siRNAs per cistron

**Figure 4:**
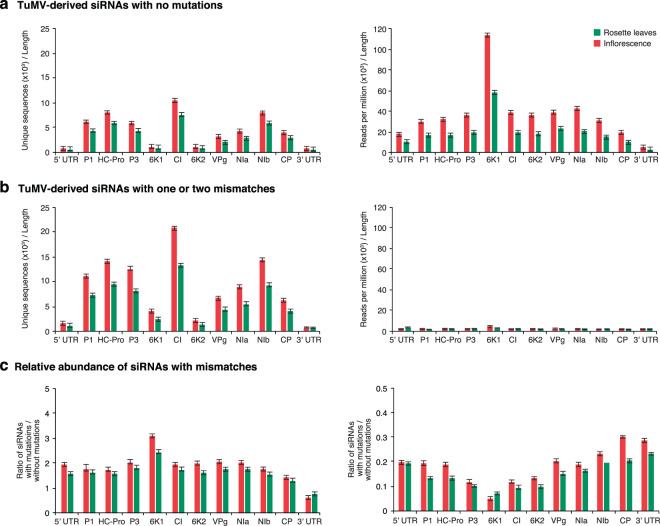
Accumulation of 21- to 24-nt virus-derived siRNAs per cistron in TuMV in inoculated Arabidopsis rosette leaves and systemically infected inflorescence. Values are the average and standard error of two biological replicates as in Fig. 1. Abundance was normalized to reads per million and to the length of the cistron. (**a**) Accumulation of siRNAs with no mutations. (**b**) Accumulation of unique sequences with one or two mismatches. (**c**) Relative abundance of siRNAs expressed as the ratio of siRNAs with one or two mismatches to siRNAs with no mutations.

Virus-derived siRNAs originate from the entire genome in both polarities (Fig. 2). However, not all parts of the genome are equally represented. Virus-derived siRNAs were mapped to individual cistrons, the 5’ UTR, and the 3’ UTR, and their abundance normalized to the length of the cistron. In all cistrons, unique siRNAs with mutations were more frequent than virus-derived siRNAs with no mutations. Both, in inoculated rosette leaves and in systemically infected inflorescence, CI accumulated the highest number of unique siRNAs with mutations, and 6K1 accumulated the highest number of siRNAs reads with mutations (Fig. 4a,b). 6K1 and CP were the cistrons with the highest relative accumulation of unique siRNAs, and siRNA reads, respectively, with mutations (Fig. 4c). All other cistrons accumulated similar relative amounts of siRNAs with mutations (Fig. 4c), suggesting that nucleotide variation in virus-derived siRNAs does not map to particular areas of the genome.

### Antiviral silencing imposes selection pressure

**Figure 5:**
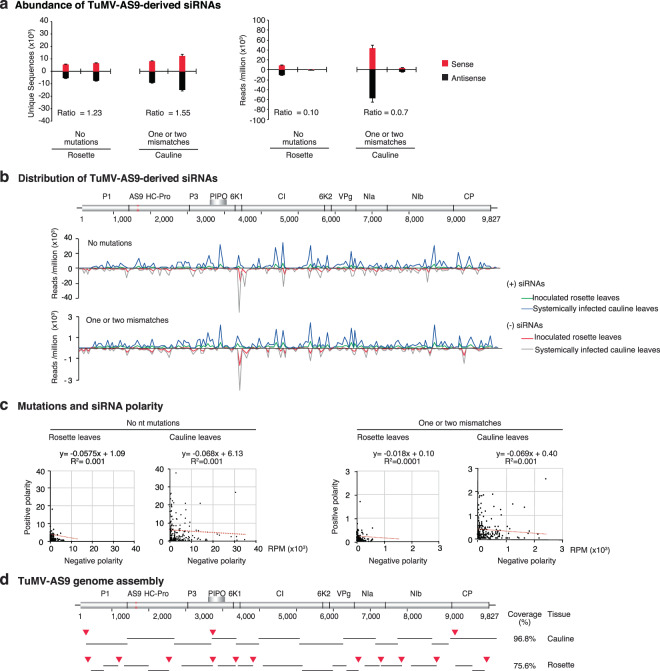
Genome-wide distribution of siRNAs derived from suppressor-deficient TuMV-AS9 and *de-novo* genome assembly. siRNAs 21- to 24-nt were pooled and classified based on the number of mismatches. Numbers represent average and standard error from two biological replicates from inoculated rosette leaves (7 dpi) and systemically infected cauline leaves (15 dpi) of *ago2-1* single mutant Arabidopsis. Number of reads were normalized to reads per million. (**a**) Unique sequences and their abundance by polarity. The ratio of siRNAs with mismatches to siRNAs with no mutations is indicated for each tissue. (**b**) Genome-wide distribution of virus-derived siRNAs by match class, polarity, and tissue. (**c**) Correlation between siRNAs of positive and negative polarity, by match class and tissue. (**d**) *De-novo* assembly of the TuMV-AS9 genome using siRNAs with no mutations. Coverage is expressed as percent of the genome. Red arrow heads point to gaps in the assembly.

We partially removed gene silencing by using *ago2–1* single mutant Arabidopsis plants and silencing suppressor-deficient TuMV-AS9^27^. TuMV-AS9-derived siRNAs were profiled from mechanically inoculated rosette leaves, and systemically infected cauline leaves (Fig. 5). Virus-derived siRNAs originated from the entire genome in both polarities (Fig. 5a,b), and the spatial distribution was not symmetrical (Fig. 5b,c).

In mechanically inoculated rosette leaves and systemically infected cauline leaves, TuMV-AS9-derived siRNAs without mutations were more abundant (with a ratio of 0.10) than siRNAs with mutations (0.07) (Fig. 5a). Interestingly, in mechanically inoculated rosette leaves, the number of unique siRNA with mutations was higher (1.23 times) than the number of unique siRNAs without mutations. In systemically infected cauline leaves of the same plants, the number of unique siRNA with mutations was higher (1.55 times) than the number of unique siRNAs without mutations (Fig. 5a). TuMV-AS9 genome assembly showed that in systemically infected cauline leaves contigs covered 96.8% (with 3 gaps), while contigs from mechanically inoculated rosette leaves covered only 75.6% (with 10 gaps) (Fig. 5d). These results are in contrast with the observed for wild type TuMV in wild type Arabidopsis, in which virus-derived siRNA populations were more diverse in locally infected leaves that in systemically infected inflorescence (Fig. 1b). The difference supports the hypothesis that antiviral gene silencing imposes purifying selection on the virus.

**Figure 6:**
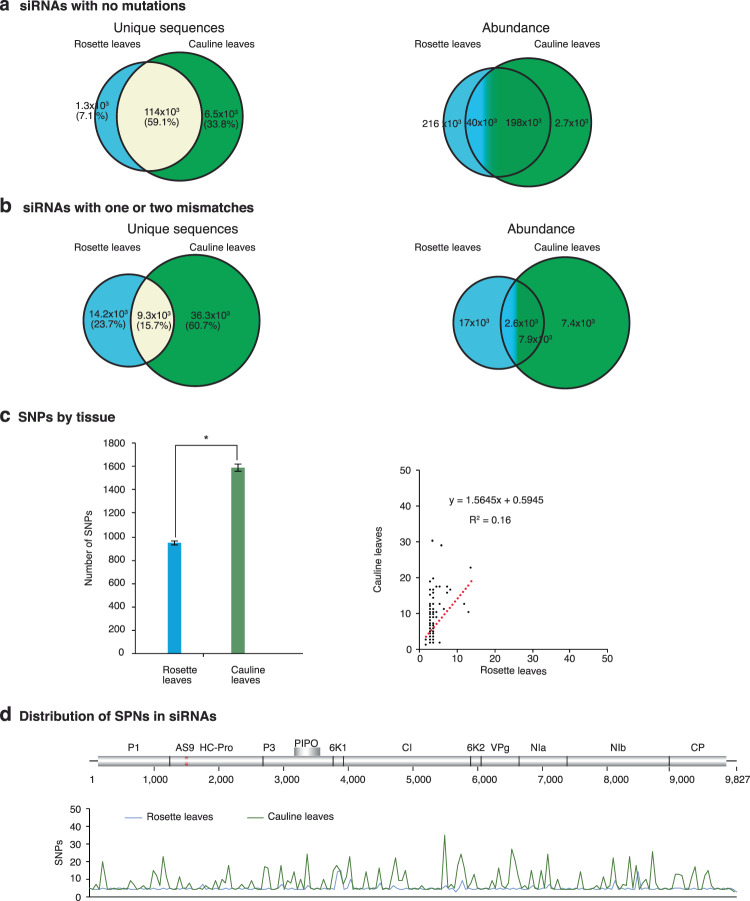
Comparison of suppressor-deficient TuMV-AS9-derived siRNAs in inoculated rosette leaves and systemically infected cauline leaves of *ago2-1* single mutant Arabidopsis. 21- to 24-nt siRNAs were pooled and correspond to samples in Fig. 5. Values are the average of two biological replicates. Unique sequences were counted per tissue and their abundance normalized to reads per million. Virus-derived siRNAs with (**a**) no mutations and (**b**) with one or two mismatches. (**c**) Accumulation of single nucleotide polymorphisms (SNPs) in virus-derived siRNAs in inoculated rosette leaves, in systemically infected inflorescence, and their correlation. The asterisk (*) indicates significant difference (p-value < 0.001). (**d**) Genome-wi**d**e distribution of single nucleotide polymorphisms in virus-derived siRNAs estimated in a 50-nt window.

Tissue-specific and common TuMV-AS9-derived siRNAs were detected in mechanically inoculated rosette leaves and in systemically infected inflorescence (Fig. 6a,b). Consistent with the higher accumulation of TuMV-AS9 in systemically infected cauline leaves than in mechanically inoculated rosette leaves^27^, the total number of siRNAs with mutations was higher in cauline leaves than in inoculated rosette leaves (Fig. 6c). In TuMV-AS9-derived siRNAs, mutations accumulated in all parts of the genome both in local and systemically infected leaves, and there was no correlation with the genome-wide distribution of mutations across tissues (Fig. 6c,d). These results support the hypothesis that new mutations are introduced in systemically infected tissue.

Per cistrons in the TuMV-AS9 genome, CI accumulated the highest number of unique sequences with mutations, and 6K1 accumulated the highest number of siRNA reads. Relative accumulation of unique siRNAs, and siRNA reads, with mutations was approximately similar (Supplementary Fig. 1). This result is similar to the observed in wild type plants infected with wild type TuMV (Fig. 4). Accordingly, there are virus intrinsic properties that contribute to the distribution of virus-derived siRNAs per cistron. However, nucleotide variation in virus-derived siRNAs does not map to particular areas of the genome.

### Mutations in small RNAs derived from *Wheatstreakmosaicvirus*

We profiled siRNAs in *Triticum aestivum* (a monocot) infected with *Wheat* *streak mosaic virus* (WSMV, a tritimovirus). Profiles were generated from small RNA libraries made from cultivar Arapahoe at 18 °C and 27 °C and one from Mace at 27 °C^40^ that were mechanically inoculated with SWMV. Cultivars Arapahoe and Mace have contrasting tolerance to WSMV. Arapahoe is susceptible at 18 °C and at 27 °C while Mace is resistant to WSMV at temperatures below 19 °C^41^.

WSMV-derived siRNAs were predominantly 21- to 24-nt long. On average, unique siRNAs with mutations were more frequent (2.77 fold) than unique siRNAs with no mutations. However, siRNAs without mutations were more abundant (31.4 -fold) than siRNAs with mutations and no strand bias was detected (Supplementary Fig. 2).

**Figure 7:**
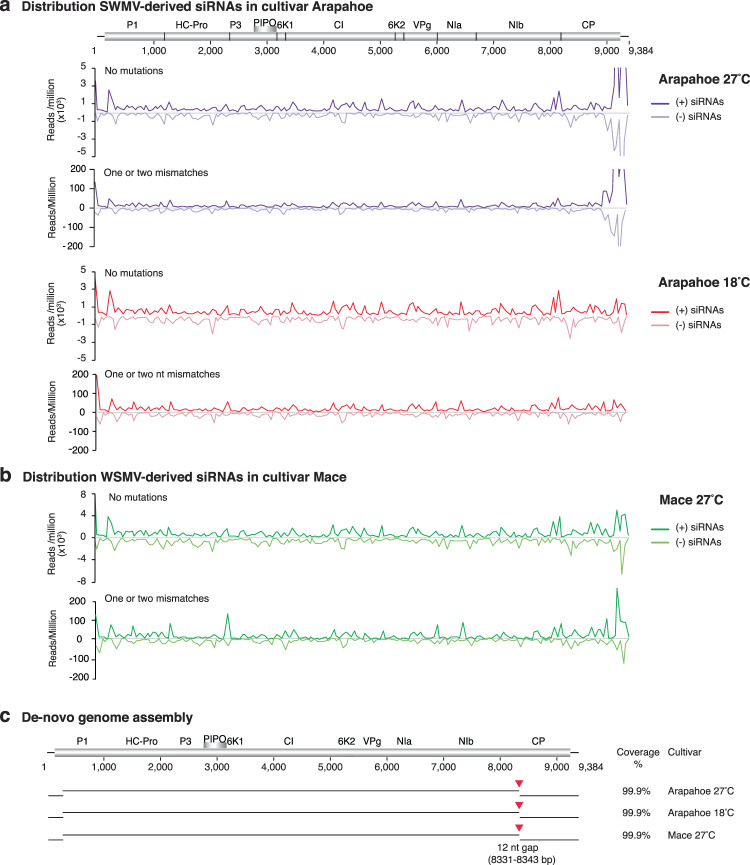
Genome-wide distribution of *Wheat streak* *mosaic* *virus* (WSMV)-derived siRNAs and *de-novo* genome assembly. SiRNAs (21- to 24-nt) were pooled and correspond to two contrasting cultivars, Arapahoe (susceptible) at 18 °C and 27 °C and Mace (susceptible at high temperature) at 27 °C. Abundance was normalized to reads per million. (**a**) Genome-wide distribution of WSMV-derived siRNAs in cultivar Arapahoe, and (**b**) cultivar Mace. (**c**) *De-novo* assembly of the WSMV genome using siRNAs with no mutations. Genome coverage is indicated in percent. Red arrow heads point to gaps (12 nt) in assembly, which map to the coat protein from nt 8331 to 8343.

In cultivar Arapahoe, virus-derived siRNAs with or without mutations were more abundant at 27 °C than at 18 °C (Supplementary Fig. 3a). In cultivar Mace, siRNAs containing mutations were more abundant than in Arapahoe. Genome-wide distribution of siRNAs showed abundant accumulation of WSMV-derived siRNAs in both cultivars (Fig. 7). In Arapahoe at 27 °C the profile showed that near the C terminal part of the CP cistron, siRNAs are more abundant than in the rest of the genome (Fig. 7a). This pattern was not present in Arapahoe at 18 °C (Fig. 7a).

Using siRNAs without mutations, the WSMV genome was assembled to 99.9%. A gap of 12-nt was detected near the N terminus of the CP cistron in both cultivars and temperatures (Fig. 7c). This gap maps to the hypervariable area in WSMV and to a part previously described as dispensable^10,40^.

The highest number of single nucleotide polymorphisms was observed in cultivar Mace at 27 °C followed by susceptible cultivar Arapahoe at 27 °C and 18 °C (Supplementary Fig. 3a). The distribution of siRNAs harboring mutations was similar between cultivars and between temperatures within the same cultivar (Supplementary Fig. 3b–d).

Accumulation of virus-derived siRNAs per WSMV cistrons showed higher accumulation in the 3′ UTR, the 5′ UTR, and the CI cistron (Supplementary Fig. 4a). CI accumulated the highest number of siRNAs with mutations (Supplementary Fig. 4b). However, the relative accumulation of siRNAs with mutations was similar in all cistrons and in both cultivars (Supplementary Fig. 4c). This pattern partially overlaps with the observed for TuMV in Arabidopsis (Fig. 4) and supports the hypothesis that the mechanisms that introduce mutations in virus-derived siRNAs is conserved across monocot and dicot plants. However, there are virus intrinsic properties that contribute to the distribution of virus-derived siRNAs per cistron, which might interact with the host and environmental factors.

### Mutations in small RNAs derived from *Papayaringspotvirus*

**Figure 8:**
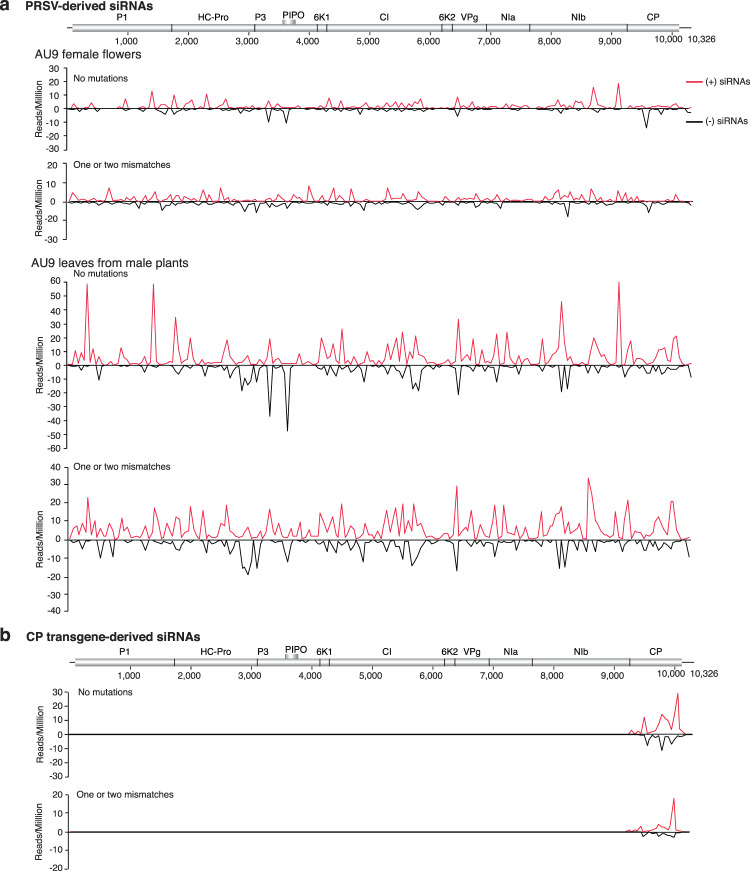
Genome-wide distribution of *Papaya* *ringspot* *virus* (PRSV)-derived siRNAs. Abundance of siRNAs 21 to 24-nt was normalized to reads per million. (**a**) PRSV-derived siRNAs in non-transgenic cultivar AU9. (**b**) Transgene-derived siRNAs in leaves from transgenic plants cultivar SunUP, expressing a coat protein transgene.

We profiled siRNAs from non-transgenic papaya plants cultivar AU9 (flowers and leaves) mechanically inoculated with *Papaya* *ringspot* *virus* (PRSV). PRSV-derived siRNAs, predominantly 21- to 24-nt, with no mutations and with one or two mutations were detected from the entire viral genome in both polarities (Fig. 8a). Virus-derived siRNAs were more abundant in leaves than in flowers, and siRNAs of positive polarity were more abundant than siRNAs of negative polarity (Supplementary Fig. 5a,b). Unique siRNAs with one or mutations were more abundant (1.4 fold) than siRNAs with no mutations. Virus-derived siRNAs with no mutations were more abundant (6.6 fold) than siRNAs with mutations (Supplementary Fig. 5a).

A larger number of siRNAs with mutations was detected in leaf samples than in flowers of non-transgenic cultivar AU9 infected with PRSV (Supplementary Fig. 5a). These samples displayed discrete sources of mutations along the PRSV genome (Supplementary Fig. 6a-b). CI was the cistron with the most unique sequences and 6K1 was the cistron with the largest number of siRNAs reads with no mutations (Supplementary Fig. 7a,b). Citrons coding for CI, NIb, P1, and HC-Pro accumulated the highest number of unique sequences and reads with mutations (Supplementary Fig. 7b). The relative accumulation of siRNAs with mutations was similar in all cistrons (Supplementary Fig. 7c).

### Cellular RNA polymerases introduce mutations

Results described above for TuMV, WSMV, and PRSV showed that virus-derived siRNAs harbor mutations originating from two possible sources: viral RNA-dependent RNA polymerases responsible for virus replication and cellular RNA-dependent RNA polymerases responsible for gene silencing amplification. Current profiling methods cannot separate these two populations. We used a genetic approach to separate the sources of mutations in virus-derived siRNAs. Based on PRSV, we profiled siRNAs derived from transgenic plants cultivar SunUP expressing the PRSV coat protein (CP) as a transgene. Compared to PRSV-derived siRNAs detected in PRSV-infected plants, there were several key differences. Transgene-derived siRNAs exclusively mapped to the coat protein (Fig. 8b), siRNAs of positive polarity were more abundant than those of negative polarity (Supplementary Fig. 5b). Interestingly, mutations were detected in siRNAs derived from the coat protein transgene (Fig. 8b). Mutations in transgene-derived siRNAs of negative polarity are specially informative. There is no PRSV replication in transgenic plants expressing the coat protein as a transgene. Accordingly, mutations in transgene-derived siRNAs are independent from virus replication and are introduced by cellular processes. The coat protein transcript is generated by cellular DNA-dependent RNA polymerase IV. Accordingly, mutations would be detected in siRNAs of positive polarity. However, nucleotide substitutions in siRNAs of negative polarity might be introduced by cellular RNA-dependent RNA polymerases during dsRNA synthesis. Mutations detected in transgene-derived siRNAs of negative polarity (Fig. 8b) unambiguously show that cellular RNA polymerases introduce mutations during transcription and antiviral gene silencing amplification.

### Contrasting pattern of mutations in genomic RNA and virus-derived siRNA

**Figure 9:**
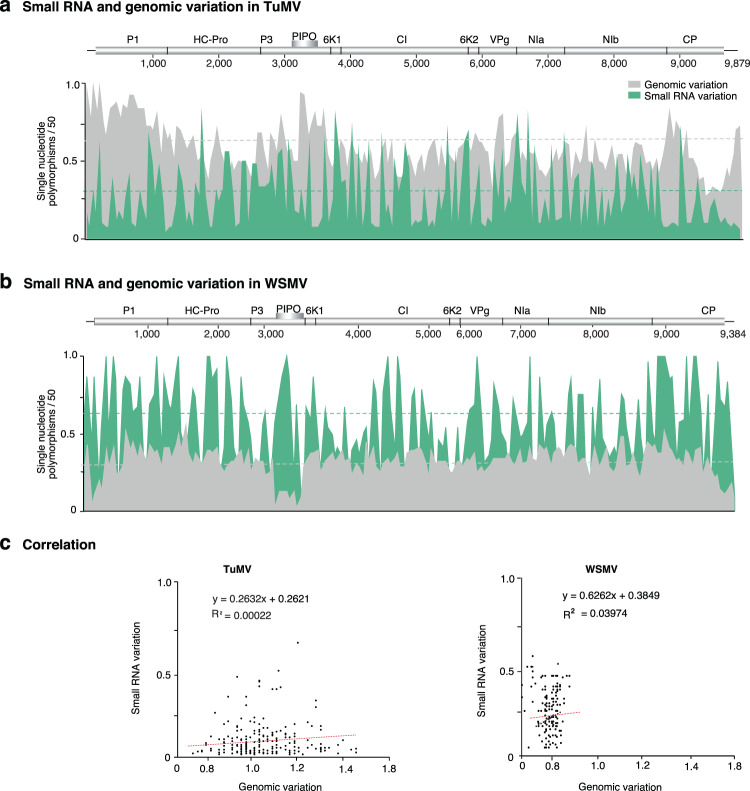
Comparison between the accumulation of mutations in virus-derived small RNAs and in the genome. Using full genome sequences or small RNAs, single nucleotide polymorphisms were estimated and normalized to a 50-nt window. The average and a 99% confidence interval are represented by a horizontal line. (**a**) Turnip mosaic virus. SiRNA variation in inflorescence is as in Fig. 2a. (**b**) Wheat streak mosaic virus. SiRNA variation in cultivar Arapahoe at 18 °C is as in Fig. 7a. (**c**) Correlation between genomic and siRNAs variation.

The potyviral genome harbors hypervariable areas^10^. In TuMV and WSMV, mutations in virus-derived siRNAs did not overlap with hypervariable genomic areas (Fig. 9). In TuMV, genomic variation preferentially accumulates in hypervariable genomic areas within cistrons coding for P1, HC-Pro, NIb, and CP. However, within those cistrons, areas with high genomic RNA variation accumulated low amounts of mutations in virus-derived siRNAs (Fig. 9a). In contrast, 6K1 and CI, which are not hypervariable, accumulated higher amounts of virus-derived siRNAs with mutations. Consistent with this difference, there was no correlation between genomic variation and virus-derived siRNA variation (Fig. 9a). Similarly, in WSMV, there was no correlation between genomic variation and virus-derived siRNA variation (Fig. 9b,c).

### Nature of nucleotide substitutions in virus-derived small RNAs

Nucleotide substitutions were classified into transitions, transversions, and normalized to the total number of reads per library. Collectively, transitions and transversion represented 37.4% and 62.6%, respectively. These numbers are close to the expected based on a random distribution of transitions (33.3%) and transversions (66.7%). However, the A to G transition was the most abundant (12.1%) nucleotide substitution and was detected at a frequency higher than the expected randomly (8.3%) (Supplementary Fig. 8a,b). Two-way cluster analyses showed that A to G transition was the most abundant nucleotide substitution across TuMV, PRSV, and WSMV, both in locally inoculated leaves and in systemically infected tissue (Supplementary Fig. 8c). Transversions C to A, T to G, and T to A, and A to C occurred at frequencies higher than expected randomly, across viruses and tissues analyzed (Supplementary Fig. 8c).

## Discussion

Infected plants accumulate virus-derived small interfering RNAs^21,24^ (Fig. 1). Based on the source, virus-derived siRNAs are classified into primary and secondary. Primary virus-derived siRNAs are derived from initial triggers, such as replication intermediates and fold-back structures in viral genomic RNA. Secondary virus-derived siRNAs come from dsRNA synthesized by cellular RNA-dependent RNA polymerases during gene silencing amplification^3^. This mechanism of siRNA biogenesis predicts that mutations in primary virus-derived siRNAs are introduced by the viral replicase during transcription or genomic RNA synthesis. In contrast, mutations in secondary virus-derived siRNAs are introduced by cellular RNA-dependent RNA polymerases. Current sequencing and biochemical methods cannot separate primary from secondary virus-derived siRNAs. Before this study, mutations in virus-derived siRNAs had not been characterized. In this study, we showed that, respect to the reference genome, mismatches in virus-derived siRNA are bona fide mutations, and we profiled mutations in siRNAs derived from TuMV, WSMV, and PRSV.

**Figure 10:**
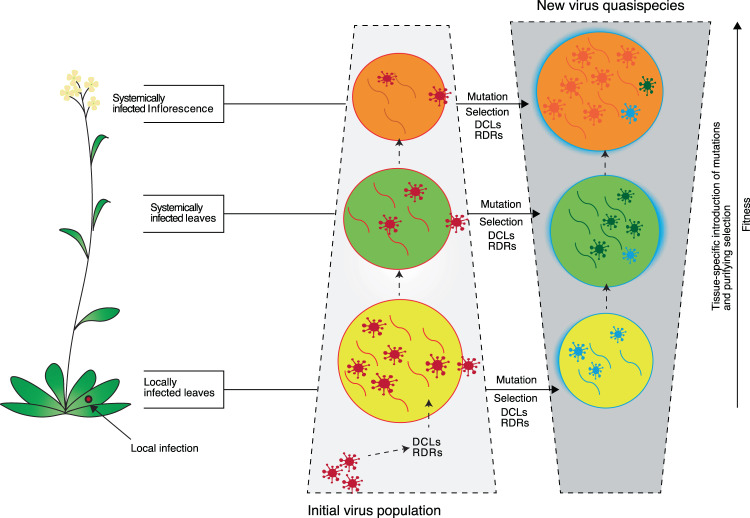
Model for the introduction of mutations in genomic viral RNA and in virus-derived siRNAs. Viral RNA-dependent RNA polymerases introduce mutations in viral RNA during replication and transcription. Cell-to-cell movement, systemic virus movement, and antiviral gene silencing impose purifying selection. During antiviral gene silencing amplification, cellular RNA-dependent RNA polymerases introduce mutations.

In TuMV-infected Arabidopsis, comparison across tissues of the same plants showed that the viral population is more diverse early in infection in mechanically inoculated leaves than in systemically infected tissue (Figs. 1 and 2). Additionally, within virus-derived siRNAs, there were mutations exclusive to mechanically inoculated rosette leaves, and mutations exclusive to systemically infected inflorescences (Fig. 3). These observations are consistent with cell-to-cell and systemic movement imposing purifying selection pressure on virus populations, and new mutations arising in systemically infected tissue (Fig. 10).

In suppressor-deficient TuMV-AS9 infecting silencing-compromised *ago2–1* mutant plants, the effect of gene silencing was partially removed. Profiles showed that virus-derived siRNAs are more diverse both in mechanically inoculated rosette leaves and systemically infected cauline leaves (Fig. 5) than in equivalent samples from wild type TuMV infecting wild type plants (Fig. 1). These difference supports the hypothesis that antiviral gene silencing imposes purifying selection on the virus (Fig. 10).

In transgenic plants expressing the PRSV coat protein, mutations were detected in siRNAs of positive and negative polarity (Fig. 8b), in the absence of PRSV replication. The coat protein transgene is expressed by transcription. Thus, mutations in siRNAs of positive polarity might be introduced by cellular DNA-dependent RNA polymerase IV during mRNA transcription. In contrast, mutations in siRNAs of negative polarity (Fig. 8b) result from gene silencing amplification mediated by cellular RNA-dependent RNA polymerases (Fig. 10).

Introduction of mutations in virus-derived siRNAs by cellular RNA-dependent RNA polymerases provides an explanation of several phenomena in plant-virus interactions. Since the generation of the first siRNA profiles^17,30,31^, there was a lack of correlation between the abundance of positive- and negative-strand genomic RNA and the abundance of positive- and negative-strand virus-derived siRNAs (Fig. 1b). In virus-derived siRNAs, unique sequences containing mutations are numerous and each was present at low abundance (Fig. 1a); and there is an asymmetry in the genomic distribution of mutations in virus-derived siRNAs of positive and negative polarity (Fig. 2).

Genome-wide variation detected using full-length RNA sequences, was not a predictor of variation observed using virus-derived siRNAs (Fig. 9). This difference might be in part explained by the contribution of cellular RNA-dependent RNA polymerases to the generation of mutations in virus-derived siRNAs. While genome-wide variation is attributed to viral RNA replication proteins^13^, variation in virus-derived siRNAs has at least two sources: viral RNA replication proteins and cellular RNA-dependent RNA polymerases. It is tempting to speculate that dsRNA generated by cellular RNA-dependent RNA polymerases might recombine with genomic viral RNA. The main obstacle for this event is the processing of dsRNA into siRNAs by dicer-like proteins (DCL). However, some viral silencing suppressors, such as the *turnip*  *crinkle* *virus* coat protein, compromise DCL proteins^42^.

Several features of the virus-derived siRNA profiles were similar in TuMV, WSMV, and PRSV: lack of strand bias, higher accumulation of virus-derived siRNAs in the CI and 6K1 cistrons, higher frequency of the A to G transition and the C to A transversion. These observations support the model that the mechanisms that introduce mutations in virus-derived siRNAs is conserved across monocot and dicot plants, and possibly across viruses. Additionally, there are virus intrinsic properties that contribute to the distribution of virus-derived siRNAs per cistron, which might interact with the host and environmental factors, as observed in cultivars with a genetic difference in temperature-dependent difference in susceptibility to WSMV (Supplementary Fig. 4).

## Methods

Computational analyses were done on high-performance computing nodes at the University of Nebraska-Lincoln Holland Computing Center (https://hcc.unl.edu/). Custom scripts are available upon request.

### Small RNA sequence data and processing

Small RNA sequence data sets used in this study are available in Gene Expression Omnibus. *Turnip mosaic virus* (TuMV)-derived siRNAs were profiled from mechanically inoculated rosette leaves and systemically infected inflorescence of wild type *Arabidopsis thaliana* (Arabidopsis). Data sets correspond to accession number GSE64911. Inoculated rosette leaves (libraries 77 and 78) were collected at 5 days post-inoculation (dpi), and systemically infected inflorescence (libraries 69 and 70) were collected at 10 dpi. Single *ago2-1* Arabidopsis mutants were inoculated with suppressor deficient TuMV-AS9. Samples from inoculated rosette leaves (libraries 21 and 22) and systemically infected cauline leaves (libraries 29 and 30 in GSE64911) were collected at 7 dpi and 15 dpi, respectively. As related to antiviral gene silencing response, these libraries were described before^27^.

Small RNA libraries from wild type and transgenic *Carica papaya* were described in^43^. In non-transgenic cultivar AU9 infected with *Papaya ringspot virus* (PRSV) samples were taken from flowers of female plants (accession GSM712526) or leaves from male plants (accession GSM712527). Transgenic cultivar SunUp expressed the PRSV coat protein as a transgene and small RNA libraries were made from female leaf samples (accession GSM712525).

Wheat cultivars Arapahoe and Mace have a contrasting response to *Wheat streak mosaic virus* (WSMV). Mace is resistant to WSMV at 18°C and susceptible at 27 °C. Arapahoe is susceptible both at 18°C and at 27°C. Plants were mechanically inoculated, and small RNA libraries from systemically infected leaves were made for both cultivars at both temperatures. Accession numbers were as follows: Arapahoe at 27°C (GSM1306038), Arapahoe at 18°C (GSM1306039), and Mace at 27°C (GSM1306040), as described^40^.

Raw data for TuMV and WSMV was in fastq format. TuMV libraries were demultiplexed using Libparse.pl^32^. Read quality of the fastq files were checked with FastQC. In libraries from wheat, adaptors were removed using adapt tool^44^ (version 0.9.5) with the following parameters: a maximum error rate of 15% (-e 15), base trimming when the Phred-like quality score was lower than 45 (-q 45), and iterating up to three times (-n 3). The PRSV data was available as in text format and libraries were normalized to reads per million^43^.

High quality reads from each of the samples were mapped to their corresponding reference genome: TuMV (NC_002509.2), PRSV (NC_001785.1) and WSMV(NC_001886.1) using Bowtie2 with two mismatches allowed^45^. Downstream analyses were performed using custom bash and Perl scripts.

### Unique virus-derived siRNAs and their abundance

From Bowtie2, the resulting SAM files were used to filter virus-derived siRNAs into fasta files based on the following criteria: match class (perfect match, one or two mismatches to the reference viral genome), polarity (sense or antisense), and size class (18 to 30 nt). Unique sequences and their abundance (number of reads) were determined for each size class and mismatch group. Per library, abundance of siRNAs was normalized to Reads Per Million (RPM)^32^. Unique sequences measuring 21- to 24-nt were captured in a fasta file. Reads containing ambiguous bases were discarded.

### Mutations in virus-derived siRNAs

To characterize mutations in virus-derived small RNAs, single nucleotide variants were determined in sequences that were 21- to 24-nt long. The fasta file generated by parsing the SAM file was used as input. Sequences were mapped the genome using the Burrows-Wheeler Aligner (BWA) (http://bio-bwa.sourceforge.net/)^46^, allowing one or two mismatches. The alignment file generated by BWA (SAM) was used as input for variant discovery using SAMtools^47^ and recorded in Variant calling format (VCF)^48^. High-confident variant sites were filtered based on the following criteria: mapping quality >50 (-C in Samtools calling), base quality/base alignment quality >20 (-Q in Samtools calling),>10 reads (-d in Samtools filter) covering each site. In parallel, Sam2Tsv (http://lindenb.github.io/jvarkit/Sam2Tsv.html) tool was used to characterize the variants where the.SAM files (from BWA output) of each libraries was used as input along with the virus reference genome^49^. The resulting output (as tsv file) was parsed and examined to categorize the variants as Transitions (Ts) or Transversions (Tv). To maximize specificity, high-quality variants were selected from the intersection of those identified both by Samtools and Sam2Tsv. The frequency of each transition and transversion was determined using in-house scripts. A heat map was generated and analyzed using the frequency (Ts /Tv) across the tissues/experiments of virus infected plants employing hierarchical clustering using the ClustVis package in R (Wickham *et al*., 2013).

### Genome-wide distribution of virus-derived siRNAs

Virus-derived siRNAs (21 to 24-nt long) from the fasta file were separated into perfect matches (no mutations) and mismatches (one or two nucleotide substitutions, insertions or deletions) for both sense and anti-sense polarity. The abundance of those siRNAs was determined on a 50-nt window using a shell script and maps to the virus genome, generating a genome-wide distribution.

### ***De novo*assembly of virus genome from virus-derived siRNAs**

Small RNAs of 21- to 24-nt with no mutations, from two biological replicates, were combined as a single fasta file, and used as input for *de-novo* genome assembly using Velvet 0.7.31 (https://www.ebi.ac.uk/~zerbino/velvet/)^50^. Different kmer options (13,15 and 17) were optimized, where k-mer of 15 was found to produce larger contigs with best assembly outcomes. The quality of the genome assembly (% coverage) was checked with the QUAST (http://quast.bioinf.spbau.ru/)^51^. Viral contigs were mapped to the reference genome.

### Tissue-specific siRNA variants

Virus-derived siRNAs (21- to 24-nt siRNAs) with or without mutations were analyzed separately, and classified as tissue-specific or common using Venny tools^52^. In Arabidopsis, siRNAs (fasta file) from rosette leaves were compared to siRNAs from cauline leaves or inflorescence. Subsequently, abundance (number of reads per million) of siRNAs for common and unique sequences between tissues were captured by parsing the SAM files of the corresponding libraries using in house bash scripts.

### Accumulation of virus-derived siRNAs per cistron

Accumulation of virus-derived siRNAs (21- to 24-nt) was determined for each cistron, the 5′UTR, and 3′UTR using the SAM file. For each region in the viral genome, siRNAs were binned using a custom bash script. The number of unique sequences and their abundance (reads per million) determined for perfect matches and mismatches, separately. The number of unique sequences and their abundance was normalized to the length of the cistron or UTR.

## Supplementary information

Supplemental information.

Supplemental information 2.

Supplemental information 3.

Supplemental information 4.

Supplemental information 5.

Supplemental information 6.

Supplemental information 7.

Supplemental information 8.

Supplemental information 9.
