# Supplementary material for: Mutations in virus-derived small RNAs
Source: Sci Rep. 2020 Jun 12;10:9540. doi: 10.1038/s41598-020-66374-2 (PMC7293216; doi:10.1038/s41598-020-66374-2)
Supplement: Supplementary file 1 — Supplemental information. [file 41598_2020_66374_MOESM1_ESM.docx]

**Mutations in virus-derived small RNAs**

Deepti Nigam, Katherine LaTourrette and Hernan Garcia-Ruiz*

Department of Plant Pathology and Nebraska Center for Virology, University of Nebraska-Lincoln. Nebraska, United States of America

* Correspondence:

Hernan Garcia-Ruiz

hgarciaruiz2@unl.edu

**SUPPLEMENTATY FIGURE LEGENDS**

**Supplementary Figure 1.** Abundance of 21- to 24-nt siRNAs per cistron in suppressor-deficient

TuMV-AS9.Values are the average and standard error of two biological replicates as in Fig 5. Number of unique sequences and their abundance (normalized to reads per million) were normalized to the length of the cistron. (**a**) Virus-derived siRNAs with no mutations. (**b**) Virus-derived siRNAs with mutations. (**c**) Abundance of siRNAs with mutations relative to siRNA with no mutations.

**Supplementary Figure 2.** Profile of WSMV derived siRNAs in two wheat (*Triticum aestivum*) cultivars (Arapahoe and Mace) and temperatures (18°C and 27°C). Samples are as in Fig 7. Number of unique sequences and their abundance (normalized to reads per million) were normalized to the length of the cistron. (**a**) Size distribution and abundance of 18-30 nt unique sequences with no mutations, and with one or two mismatches. (**b**) Polarity and abundance by tissue and match class. Only siRNAs of 21 to 24-nt were included. The ratio of siRNAs with mismatchest to siRNAs with no mutations is indicated for each treatment.

**Supplementary Figure 3.** Comparison of WSMV-derived siRNAs (21 to 24-nt) with mutations in two wheat cultivars. Samples are as in Fig 7. (**a**) Single nucleotide polymorphism is WSMV-derived siRNAs. (**b**) Correlation of single nucleotide polymorphism is virus-derived siRNAs in two wheat cultivars at 27°C, and in (**c**) cultivar Arapahoe at 18°C and 27°C. (**d**) Genome-wide distribution of single nucleotide polymorphism in virus-derived siRNAs.

**Supplementary Figure 4.** Abundance of 21- to 24-nt siRNAs per cistron in WSMV. Values correspond to samples in Fig 7. Number of unique sequences and their abundance (normalized to reads per million) were normalized to the length of the cistron. WSMV-derived siRNAs with (**a**) no nucleotide substitutions, or (**b**) with one or two mismatches. (**c**) Abundance of siRNAs with mutations relative to siRNA with no mutations.

**Supplementary Figure 5.** Profile of PRSV-derived siRNAs in two papaya (*Carica papaya*) cultivars. Non-transgenic cultivar AU9 was infected by PRSV. Transgenic cultivar SunUP expressed the PRSV CP as a transgene. Values are from a single biological replicate as in Fig 8. Number of unique sequences and their abundance (normalized to reads per million) were normalized to the length of the cistron. (**a**) Size distribution and abundance of 18-30 nt unique sequences with no mutations and with one or two mismatches. (**b**) Polarity and abundance by tissue and match class. Only siRNAs of 21- to 24-nt were included. The ratio of siRNAs with mismatchest to siRNAs with no mutations is indicated for each treatment.

**Supplementary Figure 6.** Comparison of 21- to 24-nt PRSV-derived siRNAs containing mutations and detected in non-transgenic plants cultivar AU9 infected with PRSV and in CP transgenic plants cultivar SunUp. Values are from a single biological replicate as in Fig 8. (**a**) Single nucleotide polymorphism is virus-derived siRNAs. (**b**) Genome-wide distribution of single

nucleotide polymorphism in virus-derived siRNAs.

**Supplementary Figure 7.** Abundance of 21- to 24-nt siRNAs per cistron in PRSV. Values are from a single biological replicate as in Fig 8. Number of unique sequences and their abundance (normalized to reads per million) were normalized to the length of the cistron. PRSV-derived siRNAs with (**a**) no nucleotide substitutions, or with (**b**) one or two mismatches. (**c**) Abundance of siRNAs with mutations relative to siRNA with no mutations.

**Supplementary Figure 8.** Nature of nucleotide substitutions in virus-derived small RNAs in three potyviruses. (**a**) Single nucleotide polymorphisms normalized to reads per million and classified as transitions and transversions. (**b**) Relative abundance, in percent, of nucleotide substitutions respect to the total. (**c**) Two-way clustering of transitions and transversions per virus and tissue.
